# Cofilin‐1 participates in the hyperfunction of myeloid dendritic cells in patients with severe aplastic anaemia

**DOI:** 10.1111/jcmm.17359

**Published:** 2022-05-17

**Authors:** Yingying Sun, Yu Zhang, Hong Yu, Huaquan Wang, Zonghong Shao, Chunyan Liu

**Affiliations:** ^1^ Department of Hematology Tianjin Medical University General Hospital Tianjin China

**Keywords:** aplastic anemia, CD4^+^ T lymphocyte, CD8^+^ T lymphocyte, cofilin‐1, mDC

## Abstract

Cofilin‐1 interacts with actin to regulate cell movement. The importance of cofilin‐1 in immunity has been established, and its involvement in a number of autoimmune diseases has been confirmed. However, its role in severe aplastic anaemia (SAA) remains elusive. Thus, the aim of the current study was to investigate the role of cofilin‐1 in patients with SAA. Flow cytometry, Western blotting and real‐time quantitative reverse transcription‐polymerase chain reaction were performed to detect the mRNA and protein expression of cofilin‐1 in myeloid dendritic cells (mDCs) from patients with SAA. The expression of cofilin‐1 was then suppressed via siRNA, and its effects on mDCs and downstream effector T‐cell function were evaluated. Cofilin‐1 expression was higher in mDCs from patients with SAA and correlated with routine blood and immune indexes. Moreover, cofilin‐1 knockdown in mDCs from patients with SAA reduced their phagocytic capacity, migration capacity, and CD86 expression through F‐actin remodelling, downregulating the stimulatory capacity of mDCs on CD4^+^ and CD8^+^ T lymphocytes. Collectively, these findings indicate that cofilin‐1 participates in the hyperfunction of mDCs in patients with SAA and that the downregulation of cofilin‐1 in mDCs from patients with SAA could be a novel treatment approach for SAA.

## 1 INTRODUCTION

Severe aplastic anaemia (SAA) is an autoimmune disease mediated by excessive activation of cytotoxic T lymphocytes (CTLs), which damage bone marrow hematopoietic stem cells.^1^, ^2^ Although it is widely accepted that the main driver of SAA is autoimmune‐mediated damage, the underlying mechanisms of excessive activation of CTLs have not been characterized. Studies have shown that in patients with SAA, T helper cell (Th) 0‐to‐Th1 polarization and Th1/Th2 imbalance cause the secretion of large amounts of type I lymphatic factors such as interleukin (IL)‐2, tumour necrosis factor (TNF)‐α, and interferon (IFN)‐γ, which facilitate CTL activation in patients with SAA. In addition, the decrease in the number and function of T regulatory (Treg) cells,^3^ which results in impaired regulation of the Th1/Th2 balance, decreased ability to monitor CTLs, and insufficient inhibition of dendritic cells (DCs),^4^ contributes to SAA pathogenesis. In our previous study, we aimed to investigate the origin of SAA immune‐related pathogenesis. The results showed that mDCs of patients with SAA are in a state of overactivation with enhanced antigen presentation.^5^ Therefore, we proposed that myeloid DCs (mDCs) might play an important role in the primary immune responses related to SAA.

To further investigate the mechanism of mDC overactivation in SAA, Liu et al.^6^ carried out a proteomic analysis of mDCs from patients with SAA. The results revealed that cofilin‐1, glucose‐6‐phosphate dehydrogenase and the pyruvate kinase enzyme M2 in mDCs from patients with SAA may contribute to mDC overactivation. In this study, we investigated the mechanism of cofilin‐1 in the overactivation of mDCs from patients with SAA. Cofilin‐1, a member of the actin‐depolymerizing factor/cofilin protein family, interacts directly or indirectly with the actin cytoskeletal system to participate in the formation, function, and recombination of the actin skeleton and regulate cell motility. Cofilin‐1 is one of the major regulators of intracellular actin remodelling. On one hand, cofilin‐1 cleaves actin filaments and causes actin depolymerization. On the other hand, it can generate new free ends and provide more actin monomer sources for the extension of the fast‐growing end of F‐actin to increase actin polymerization.^7^, ^8^ Previous studies have elucidated the role of the cytoskeletal system in autoimmune diseases, including cofilin‐1.^9^, ^10^ Actin polymerization and depolymerization, mediated by cofilin‐1, generate a time‐space‐coordinated dynamic actin cycle that is critical to various biological processes, including the immune response. It has been confirmed that cofilin‐1 plays an important role in the function of DCs. Activation of cofilin can promote phagocytosis^11^, ^12^ and inactivation of cofilin‐1 reduces the migration ability of DCs and their ability to co‐stimulate T cells.^13^ Therefore, we speculated that the involvement of cofilin‐1 in mDC activation may be of interest for the further exploration of SAA pathogenesis and therapy development. Here, we explored the role of cofilin‐1 in patients with SAA and related molecular mechanisms that might be potential targets for SAA treatment.

## 2 MATERIALS AND METHODS

### 2.1 Study subjects

Forty‐three patients newly diagnosed with SAA (25 males and 18 females), with a median age of 56 (range 6–86) years, and 15 SAA patients with complete remission (CR) (10 males and 5 females), with a median age of 31 (range 11–64) years, at the Hematology Department of Tianjin Medical University General Hospital were enrolled from January 2018 to December 2019. The diagnosis and evaluation after immunosuppressive therapy (IST) of SAA were compliant with the 2009 International AA Study Group Criteria.^14^ All patients with CR had received IST, including antithymocyte globulin, cyclosporine and eltrombopag. Fifteen healthy controls (HCs; six males and nine females), with a median age of 45 (range 15–69) years, were enrolled. There was no significant difference in participant sex or age between groups (*p* > 0.05).

Studies involving human participants were reviewed and approved by the Ethics Committee of Tianjin Medical University General Hospital (IRB2019‐KY‐098). Written informed consent to participate in this study was provided by each participant or his/her legal guardian/next of kin.

### 2.2 Flow cytometric analysis

To detect cofilin‐1 in mDCs, peripheral blood samples were collected in EDTA anticoagulant tubes. Allophycocyanin (APC)‐CD11c and phycoerythrin (PE)‐HLA‐DR (BD Pharmingen, San Diego, CA, USA) were used to label mDCs. Following cell permeabilization using the Fixation/Permeabilization Solution Kit (Invitrogen, Carlsbad, CA, USA), cofilin‐1 was labelled with a 1:100 rabbit anti‐human cofilin‐1 antibody (Abcam, Cambridge, MA, USA) and 1:1000 goat anti‐rabbit Alexa Fluor 488‐IgG secondary antibody (Proteintech, Rosemont, IL, USA). T‐cell subtypes were identified using APC‐CD3, PE‐CD4 and fluorescein isothiocyanate (FITC)‐CD8 antibodies (BD Pharmingen). Th1‐related (IL‐2, TNF‐α and IFN‐γ) and Th2‐related (IL‐4, IL‐6 and IL‐10) cytokine concentrations were measured using the Human Th1/Th2 Assay Kit (Celgene Biotech, Hangzhou, China). Plasma was obtained by centrifuging the blood samples at 300 × *g* for 5 min. The FITC Annexin V Apoptosis Kit (BD Pharmingen) was used to detect apoptosis. The cell concentration was adjusted to 1 × 10^6^/mL with binding buffer (1×). Next, 5 μL of FITC Annexin V was added to the cells, which were then incubated in the dark at room temperature for 30 min. After incubation, 5 μL of propidium iodide (PI; 50 μg/mL) was added, and the cells were then incubated in the dark for 5 min. Annexin V^−^PI^−^ cells were considered as live cells, Annexin V^+^PI^−^ cells as early apoptotic cells, Annexin V^+^PI^+^ cells as late apoptotic cells and Annexin V^−^PI^+^ cells as dead cells.

The co‐stimulatory molecules CD80 and CD86 on mDCs were detected using PE‐CD80 and PE‐CD86 (BD Pharmingen), respectively.

PE‐CD4 and FITC‐CD25 antibodies (BD Pharmingen) were used to label the surface markers of Tregs. FOXP3 was detected using Alexa Fluor 647‐FOXP3 (BD Pharmingen) after permeabilization.

FITC‐CD8 antibody (BD Pharmingen) was used to label surface CD8 before permeabilization. Intracellular perforin and granzyme B in CD8^+^ T lymphocytes were detected using PE‐perforin and APC‐granzyme B (BD Pharmingen), respectively, after permeabilization.

An isotype‐matched control antibody (IgG1; BD Pharmingen) was used. Data acquisition was performed on an FACS‐Calibur, and the acquired data were analysed using CellQuest 3.1 (Becton Dickinson, Franklin Lakes, NJ, USA).

### 2.3 In vitro culture and mDC sorting

Bone marrow samples from patients with SAA and HCs were collected in heparin anticoagulant tubes. Bone marrow mononuclear cells were isolated by density gradient centrifugation using Ficoll‐Paque Plus solution (Amersham Biosciences, Uppsala, Sweden) according to the manufacturer's instructions. Thereafter, we placed the cells in a culture medium containing 79% RPMI 1640 (Gibco‐BRL, Grand Island, NY, USA), 20% foetal bovine serum (Gibco‐BRL), and 1% penicillin–streptomycin (Hyclone, South Logan, UT, USA) at a cell concentration of 1.5 × 10^6^/mL. After incubation for 2 h, the suspended cells were discarded, and the remaining semi‐adherent cells were cultured in a medium containing 100 ng/mL recombinant human granulocyte monocyte colony stimulating factor (rhGM‐CSF) (North China Pharmaceutical Co., Hebei, China) and 25 ng/mL recombinant human (rhIL‐4) (PeproTech, Rocky Hill, NJ, USA) at 37°C with 5% CO_2_. The medium was changed, and the above‐mentioned cytokines were added every 2 days. On Day 6, 1000 U/mL rhTNF‐α (PeproTech) was added to induce mDC maturation. On Day 7, the suspended cells were collected, labelled with APC‐CD11c (BD Pharmingen) and PerCP‐HLA‐DR (BD Pharmingen), and sorted using a FACSAria flow cytometer (BD Biosciences, San Jose, CA, USA). Purity of the mDCs was measured and quantified as the percentage of CD11c^+^HLA‐DR^+^ cells among all sorted cells.

### 2.4 Western blotting

The cells were lysed using RIPA buffer (Solarbio, Beijing, China) containing 1% phenylmethanesulfonyl fluoride (PMSF) (Solarbio) and 1% phosphatase inhibitors (Solarbio). A BCA Kit (Beyotime Biotechnology, Shanghai, China) was used for protein quantification. Protein loading buffer (Solarbio) was added to make proteins fully bind to sodium dodecyl sulphate after denaturation. The protein samples were stored at −80°C until use. The proteins were separated via electrophoresis, transferred on to polyvinylidene fluoride membranes, and blocked with 5% bovine serum albumin (BSA; Solarbio). The membranes were incubated overnight in 5% BSA containing rabbit anti‐human cofilin‐1 or β‐actin antibodies (Cell Signaling Technology, Danvers, MA, USA) at a dilution of 1:1000. After washing with TBS +1% Tween (TBST; Solarbio) three times, the membranes were incubated for 1 h in 5% BSA containing goat anti‐rabbit peroxidase‐conjugated secondary antibodies (Cell Signaling Technology) at a dilution of 1:2000. Super ECL Plus Detection Reagent (Cell Signaling Technology) was used for signal detection. The detected signals were quantified using ImageJ 1.8.0.112 (U.S. National Institutes of Health, Bethesda, MD, USA).

### 2.5 RNA extraction and qRT‐PCR

TRIzol reagent (Invitrogen) was added to the cell suspension to lyse cells, and chloroform was added to collect the total RNA. Isopropanol and 75% ethanol were used to isolate and purify RNA. Complementary DNA (cDNA) was obtained by reverse transcription using the FastQuant RT (with gDNase) Kit (Tiangen, Beijing, China) according to the manufacturer's protocol. The cDNA concentration was determined using a spectrophotometer. qRT‐PCR was performed using the SuperReal PreMix Plus (SYBR Green) Kit (Tiangen), according to the manufacturer's protocol, on a Bio‐Rad PCR iQ5 device (Bio‐Rad, Hercules, CA, USA). The following primers, which were synthesized by GenePharma (Shanghai, China), were used:

forward primer of *CFL1*: 5′‐TACGCCACCTTTGTCAAGATG‐3′ (location 283–303);

reverse primer of *CFL1*: 5′‐CCTTGGAGCTGGCATAAATCAT‐3′ (location 445–424);

forward primer of *ACTB*: 5′‐TGGACATCCGCAAAGACCTGT‐3′ (location 944–964); and

reverse primer of *ACTB*: 5′‐CACACGGAGTACTTGCGCTCA‐3′ (location 1103–1083).

The thermal cycling protocol used was 95°C for 30 s, followed by 50 amplification cycles (95°C for 5 s, 54.6°C for 45 s and 70°C for 30 s), and 4°C for 10 s to terminate the reaction. The cycle threshold (Ct) was recorded, and the 2^−ΔΔCt^ method was used to calculate the relative expression level.

### 2.6 Small interfering RNA (siRNA)

mDCs were divided into control, cofilin‐1 siRNA and scrambled siRNA groups. Sorted mDCs were counted, and 2.5 × 10^5^ cells were suspended in 400 μL of culture medium and seeded in a 24‐well plate. Thereafter, 100 μL of transfection reagent mixture containing 2 μL of LipoGeneTM 2000 Star (US Everbright, Suzhou, China) was added to control cells. Alternatively, 100 μL of transfection reagent mixture containing 20 pmol of cofilin‐1 siRNA and 2 μL of LipoGene 2000 Star was added to the cofilin‐1 siRNA group. For the scrambled siRNA group, 100 μL of transfection reagent mixture containing 20 pmol of scrambled siRNA and 2 μL of LipoGene 2000 Star was added. The siRNA used were as follows: cofilin‐1 siRNA forward, 5′‐CCUCUAUGAUGCAACCUAUTT‐3′ and reverse, 5′‐AUAGGUUGCAUCAUAGAGGTT‐3′; scramble siRNA forward, 5′‐UUCUCCGAACGUGUCACGUTT‐3′ and reverse, 5′‐ACGUGACACGUUCGGAGAATT‐3′ (synthesized by GenePharma). The medium was replaced after 6 h of incubation. Transfection efficiency was determined using qRT‐PCR and Western blotting after culture for 48 and 72 h, respectively.

### 2.7 CCK‐8 assay

CCK‐8 assay was used to analyse cell proliferation. mDCs (1 × 10^5^) were resuspended in 100 μL of medium and cultured at 37°C with 5% CO_2_ for 8 h. Thereafter, 10 μL of CCK‐8 (Bimake, Houston, TX, USA) was added to each well, followed by incubation for another 2 h. The optical density (OD) of sample in each well was detected using a microplate reader (Elx800; Bio Tek, Winooski, VT, USA). The OD values of the cell‐free control wells were subtracted from the OD values of experimental wells to determine cell proliferation.

### 2.8 Phagocytosis detection

FITC‐dextran (40 kDa; Sigma‐Aldrich, St. Louis, MO, USA) was diluted to 10 mg/mL in PBS and stored at −20°C in the dark after aliquoting. A working concentration of 1 mg/mL was used. Sorted mDCs were counted, and the cell concentration was adjusted to 2.5 × 10^5^/mL. A 400‐μL cell suspension was divided into two tubes, and 22 μL of FITC‐Dextran stock solution was added into each tube. The experimental tube was placed in a 37°C incubator with 5% CO_2_, and the control tube was placed in a 4°C refrigerator. Both were incubated for 2 h. The cells were then washed twice with 4°C PBS to remove residual FITC‐dextran and subjected to flow cytometry, and the percentage of dextran‐positive cells among CD11c^+^HLA‐DR^+^ cells was determined. As both active phagocytosis and non‐specific binding between mDCs and FITC‐dextran occurred at 37°C, and only non‐specific binding occurred at 4°C, phagocytosis was quantified as the FITC‐positive rate in the 37°C experimental group minus the positive rate of FITC in the 4°C control group, excluding non‐specific binding.^15^

### 2.9 Transwell migration assay

mDCs (1 × 10^5^) were resuspended in 200 μL of serum‐free medium and added to the upper Transwell chamber. Thereafter, 500 μL of complete medium containing 6 ng/mL recombinant human RANTES (T&L Biological Technology, Beijing, China) was added to the lower Transwell chamber; the pore of the membrane was 5.0 μm. The 24‐well culture plate was placed in an incubator at 37°C with 5% CO_2_ for 8 h. The cells remaining in the upper chamber were wiped away. The cells on the outer surface of the membrane were fixed with 4% paraformaldehyde and stained with 0.1% crystal violet. The stained upper chamber was placed in a clean 24‐well culture plate and observed under an inverted microscope. The centre, top left, bottom left, top right and bottom right fields were selected for counting. Their sum represents the number of cells that migrated.

### 2.10 Immunofluorescence assay

The cell concentration was adjusted to 4 × 10^5^/mL with medium. The cells were fixed on a glass slide with 4% paraformaldehyde for 15 min, permeabilized with 0.5% Triton X‐100 (Solarbio) for 20 min, and blocked with 1% BSA for 30 min. The slides were then incubated in the dark with 100 nM tetramethylrhodamine (TRITC)‐labeled phalloidin (Solarbio) for 30 min. Antifade solution containing 4′,6‐diamidino‐2‐phenylindole (DAPI) (Solarbio) was dropped on the slide. The slides were observed and photographed under a confocal laser scanning microscope (Olympus FV1200, Tokyo, Japan).

### 2.11 Magnetic‐activated cell sorting (MACS) for CD4^+^ and CD8^+^ T lymphocytes

Peripheral blood samples were collected in heparin anticoagulant tubes, and mononuclear cells were isolated by density gradient centrifugation using Ficoll‐Paque Plus solution (Amersham Biosciences). An anti‐CD4 or anti‐CD8 monoclonal magnetic bead antibody (Miltenyi Biotec GmbH, Bergisch Gladbach, Germany) was added to the mononuclear cell suspension and incubated at 4℃ in dark for 20 min. CD4‐ or CD8‐positive lymphocytes were selected using an MS type sorting column according to the manufacturer's instructions (Miltenyi Biotec GmbH). The purity of the separated cells was determined using flow cytometry.

### 2.12 Co‐culture of mDCs and lymphocytes

Suspensions of mDCs and T lymphocytes, which were from the same individual, were adjusted to 1 × 10^6^/mL and mixed at a ratio of 1:1 in each well of 24‐well plates. CD3 and CD28 monoclonal antibodies were added to the medium at a final concentration of 400 ng/mL. The 24‐well culture plate was placed in an incubator at 37°C with 5% CO_2_ for 72 h.

### 2.13 Detection of T lymphocyte proliferation using carboxyfluorescein diacetate succinimidyl ester (CFSE)

Carboxyfluorescein diacetate succinimidyl ester is a new dye that can fluorescently label live cells. The basic mechanism is as follows: CFSE can easily penetrate cell membranes, covalently bind to intracellular proteins in live cells, and release green fluorescence upon hydrolysis. In the process of cell division and proliferation, its fluorescence intensity decreases step by step with cell division. The labelled fluorescence can be equally divided between two progenitor cells, so its fluorescence intensity is half of that of parental cells. According to this characteristic, we used CFSE to detect cell proliferation. CD8^+^ T lymphocytes were counted, and the cell concentration was adjusted to 1 × 10^7^/mL. CFSE (BD Pharmingen) storage solution (10 mM) was diluted 1:1000 with PBS to obtain CFSE reaction solution (10 µM). An equal amount of CSFE reaction solution was mixed with the CD8^+^ T lymphocyte suspension and incubated at 37°C in dark for 15 min to stain the cells. The mean fluorescence intensity (MFI) of CFSE in the CD8^+^ T lymphocytes was detected using flow cytometry.

### 2.14 Statistical analysis

SPSS version 21.0 was used for statistical analyses. Results are expressed as mean ± standard deviation. All data were normally distributed. The one‐way ANOVA was used for comparison between groups. Turkey test was used for statistical analysis between two groups. Spearman's test was used to assess the linear correlation of the results. Results with *P* value less than 0.05 were considered statistically significant.

## 3 RESULTS

### 3.1 Cofilin‐1 is upregulated in the mDCs of patients with SAA

**FIGURE 1:**
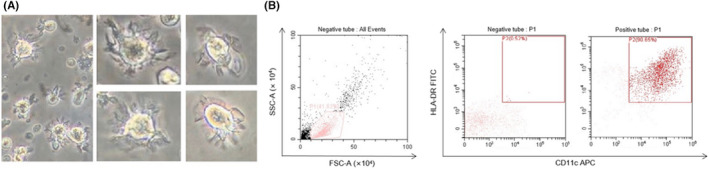
Morphology and purity of mDCs. (A) The morphology of the harvested myeloid dendritic cells (mDCs). (B) The purity of mDCs after sorting via flow cytometry reached >90%

mDCs harvested after 7 days of culture exhibited typical irregular morphology, with varying cell sizes and many dendritic protrusions of different lengths, thicknesses and densities (Figure 1A). As revealed by flow cytometry, the percentage of CD11c^+^HLA‐DR^+^ mDCs among all cultured cells was 50%–70%. After sorting, the purity of mDCs was >90% (Figure 1B).

**FIGURE 2:**
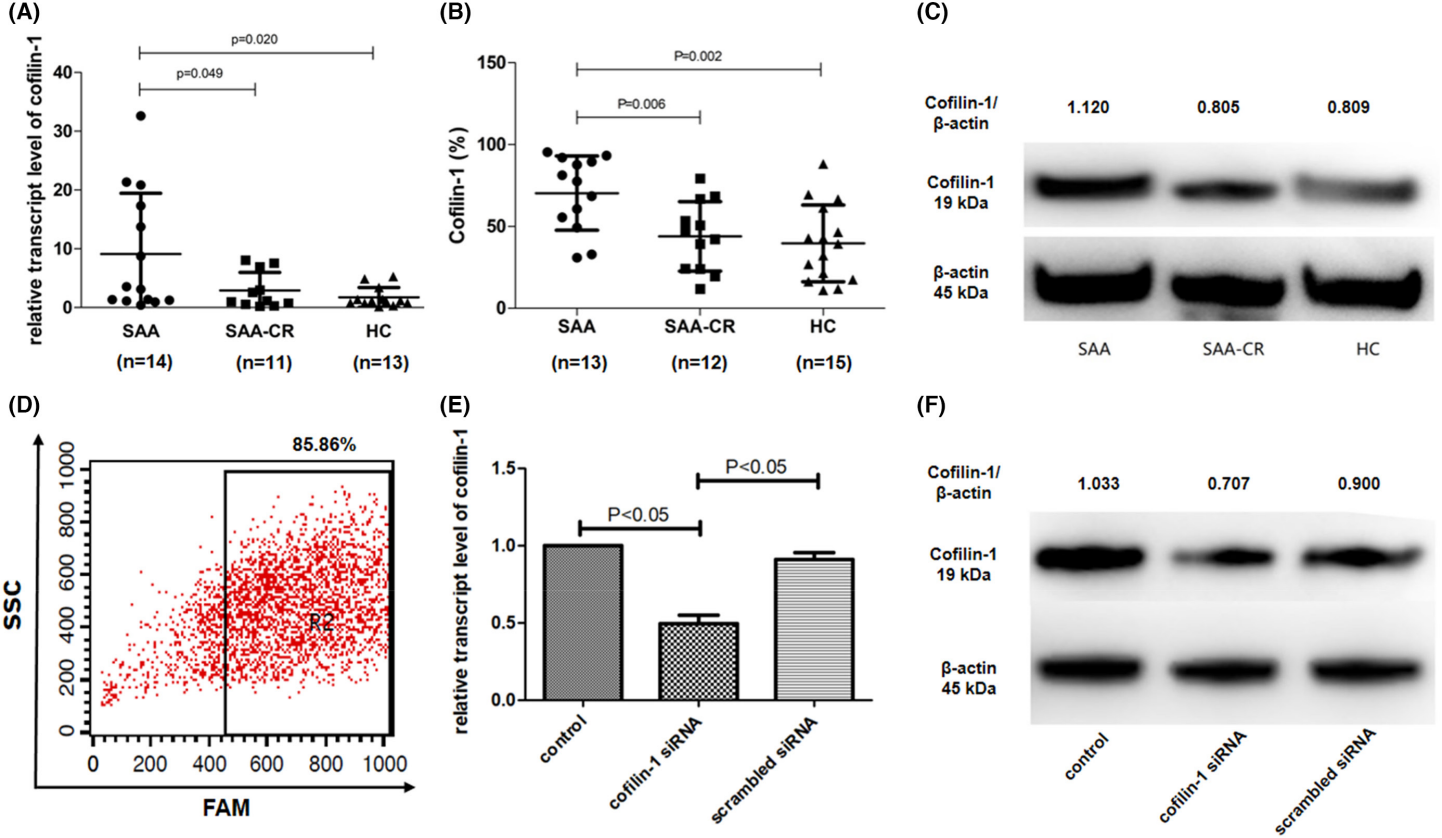
Expression level of cofilin‐1 in patients with SAA and the expression level of cofilin‐1 in mDCs from patients with SAA after cofilin‐1 siRNA. (A–C) The expression level of cofilin‐1 in mDCs from untreated patients with SAA was higher than that in mDCs from SAA patients with complete remission (CR) and healthy controls, as determined using RT‐PCR, FACS and Western blotting. (D) An FAM‐labeled siRNA oligo was used to determine the transfection efficiency using FACS. (E) The relative transcript level of cofilin‐1 in the cofilin‐1 siRNA group was 0.49 ± 0.06, which was significantly decreased compared with that in the scrambled siRNA group (0.91 ± 0.47) and control group (*n* = 3, *p* < 0.05). (F) Verification of the effect of cofilin‐1 siRNA in mDCs of patients with SAA using Western blotting

The relative transcript level of cofilin‐1 was 9.13 ± 10.32, 2.91 ± 3.08 and 1.74 ± 1.70 in mDCs from untreated patients with SAA, SAA patients with CR and HCs, respectively (Figure 2A). The relative expression of cofilin‐1 in mDCs from untreated patients with SAA was significantly higher than that in the CR (*p* = 0.049) and HC (*p* = 0.020) groups. There was no significant difference between the latter two groups (*p* = 0.275). Cofilin‐1 level in mDCs from patient with SAA (70.37% ± 22.70%) was higher than that in SAA patients with CR (43.97% ± 21.23%, *p* = 0.002) and HCs (39.65% ± 23.43%, *p* = 0.006), as revealed by FACS (Figure 2B). Western blotting confirmed these results (Figure 2C).

The level of cofilin‐1 in mDCs from patients with SAA was negatively correlated with the white blood cell count (*r* = −0.57, *p* = 0.0026), absolute value of neutrophils (*r* = −0.49, *p* = 0.0134), platelet count (*r* = −0.57, *p* = 0.0028), hemoglobin level (*r* = −0.47, *p* = 0.0192), absolute value of reticulocytes (*r* = −0.4089, *p* = 0.0424) and CD4^+^/CD8^+^ ratio (*r* = −0.62, *p* = 0.0010). In contrast, it was positively correlated with the serum concentrations of IL‐2 (*r* = 0.56, *p* = 0.0037) and IFN‐γ (*r* = 0.56, *p* = 0.0037). The cofilin‐1 level in mDCs from patients with SAA was not significantly correlated with the concentration of TNF‐α, IL‐4, IL‐6 or IL‐10.

### 3.2 Cofilin‐1 affects the function of mDCs in patients with SAA

The expression of cofilin‐1 in mDCs was successfully reduced by siRNA, as confirmed by FACS, qRT‐PCR and Western blotting (Figure 2D–F).

There were no significant differences in cell proliferation, as observed in the CCK‐8 assay, among the control (1.22 ± 0.54), cofilin‐1 siRNA (0.60 ± 0.20) and scrambled siRNA (0.86 ± 0.45) (*p* > 0.05) groups. The apoptosis analysis revealed that early apoptotic cells accounted for 7.32% ± 3.05%, 14.40% ± 10.82% and 13.15% ± 6.19% of cells in the control, cofilin‐1 siRNA and scrambled siRNA groups, respectively; late apoptotic cells accounted for 13.92% ± 10.10%, 14.91% ± 8.52% and 12.60% ± 6.86% of cells, respectively. Total apoptotic cells accounted for 21.24% ± 12.20%, 29.31% ± 18.98% and 25.75% ± 10.43% of all cells in the three groups, respectively (Figure S1, Table S1). No significant differences in apoptosis were observed between the cofilin‐1 siRNA and scrambled siRNA groups (*p* = 0.77 for early apoptosis, *p* = 0.54 for late apoptosis, and *p* = 0.63 for total apoptosis).

To assess phagocytic activity, FITC‐dextran, which is phagocytosed by mDCs and can be detected by FACS or under a fluorescence microscope, was employed. The difference in the FITC‐positive rate between 37°C and 4°C was 22.64% ± 12.53%, 40.07% ± 11.90% and 44.83% ± 17.33% in the cofilin‐1 siRNA, scrambled siRNA, and control groups, respectively. The downregulation of cofilin‐1 led to significantly lower phagocytic capacity (*p* < 0.05).

**FIGURE 3:**
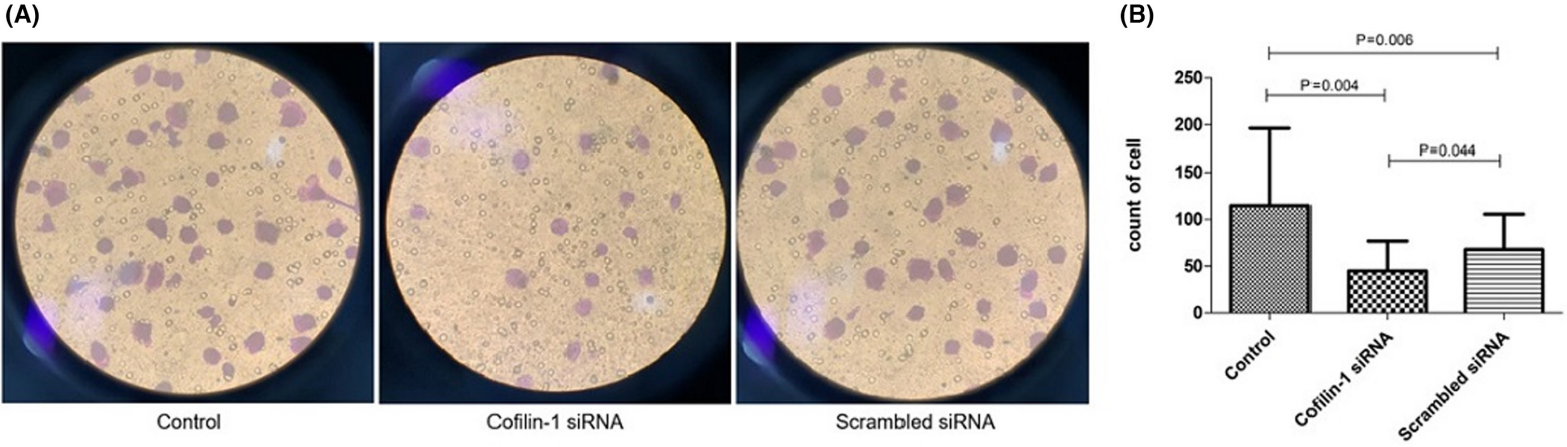
Migration of mDCs in patients with SAA was decreased after cofilin‐1 siRNA, as detected using the Transwell assay (*n* = 12). (A) The fields of Transwell from three groups viewed under a microscope (mDCs from patients with SAA). (B) The total number of cells in the five fields was lower in the cofilin‐1 siRNA group than in the other two groups (*p* < 0.05)

Thereafter, mDC migration was assessed. The total number of cells in the centre, upper left, upper left, upper right and lower right fields was 114.67 ± 81.75, 45.08 ± 31.98 and 67.75 ± 38.07 in the control, cofilin‐1‐siRNA and scrambled siRNA groups, respectively, indicating the decreased migration capacity of mDCs after cofilin‐1 knockdown (*p* < 0.05) (Figure 3A, B).

**FIGURE 4:**
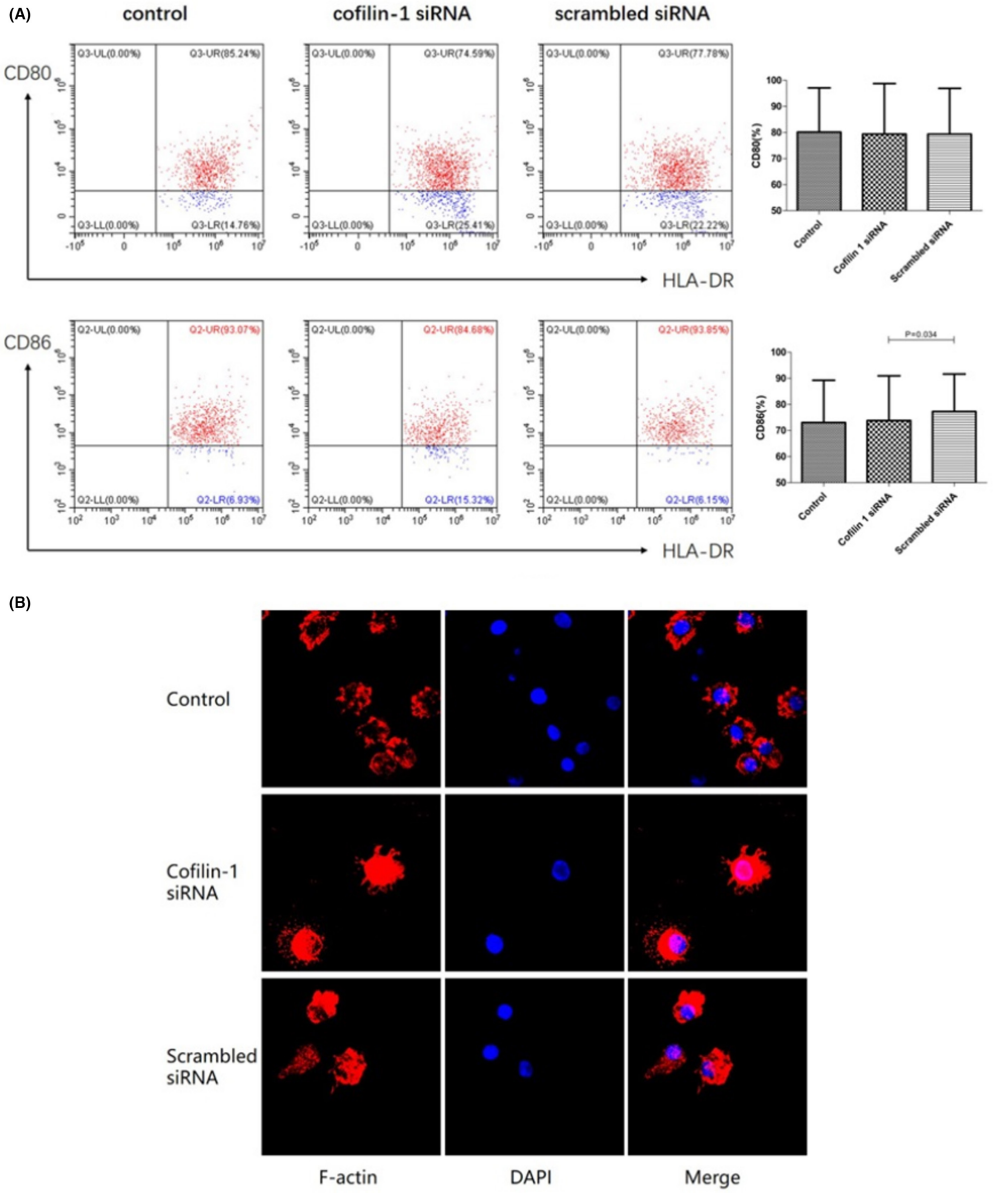
Expression of CD80, CD86 and F‐actin in mDCs from patients with SAA after the downregulation of cofilin‐1 (*n* = 12). (A) CD86 expression was lower in the cofilin‐1 siRNA group than in the scrambled siRNA group. There were no significant differences in CD80 expression among the three groups. (B) The red shade represents F‐actin and blue represents the cell nucleus. The content and distribution of F‐actin in mDCs were altered after the downregulation of cofilin‐1

CD86 expression on mDCs from the cofilin‐1 siRNA group was significantly lower than that on mDCs from the scrambled siRNA group (73.80% ± 17.18% vs. 77.26% ± 14.39%, *p* = 0.034). However, there was no difference in CD80 expression among the three groups (Figure 4A).

As cofilin‐1 is involved in cytoskeleton regulation through F‐actin, we compared the morphology and distribution of F‐actin in the groups using immunofluorescence. F‐actin was mostly distributed under the membrane in the control and scrambled siRNA groups. In contrast, both F‐actin content and cell protrusion density were increased in the cofilin‐1 siRNA group, resulting in significant remodelling (Figure 4B).

### 3.3 Cofilin‐1 in mDCs from patients with SAA participates in the activation of CD4^+^ and CD8^+^ T lymphocytes

The purity of CD4^+^ and CD8^+^ T lymphocytes obtained by MACS was over 90%. After co‐culture of mDCs and CD4^+^ or CD8^+^ T lymphocytes, which were from the same individual for 72 h, the cells proliferated and aggregated into clusters floating in the medium.

mDCs from the control, cofilin‐1 siRNA and scrambled siRNA groups were co‐cultured with CD4^+^ T lymphocytes. The concentrations of Th1‐ and Th2‐related cytokines in the co‐culture supernatant were measured, and they are shown in Tables 1 and 2. The knockdown of cofilin‐1 in mDCs led to a decrease in their ability to stimulate the production of Th1‐related (IL‐2, TNF‐α and IFN‐γ) and Th2‐related (IL‐6) cytokines by CD4^+^ T cells.

**TABLE 1:** Concentration of Th1‐related cytokines in the three groups

Group	IL‐2 (pg/mL)	TNF‐α (pg/mL)	IFN‐γ (pg/mL)
Control	235.27 ± 176.02	398.45 ± 268.48*	3248.85 ± 2176.86
Cofilin‐1 siRNA	179.48 ± 180.52*	178.08 ± 146.00* ^,^ **	2499.71 ± 2051.73* ^,^ **
Scrambled siRNA	216.32 ± 203.24	232.48 ± 157.75^**^	3020.96 ± 2340.99

* *p* < 0.05 compared with the scrambled group.

** *p* < 0.05 compared with the control group.

**TABLE 2:** Concentration of Th2‐related cytokines in the three groups

Group	IL‐4 (pg/mL)	IL‐6 (pg/mL)	IL‐10 (pg/mL)
Control	41.95 ± 59.17	837.94 ± 800.98*	281.76 ± 192.75
Cofilin‐1 siRNA	53.66 ± 77.74	357.19 ± 237.02	262.45 ± 194.54
Scrambled siRNA	66.62 ± 120.17	435.74 ± 325.01*	275.31 ± 177.78

* *p* < 0.05 compared with the scrambled group.

FOXP3 expression was observed in 74.54% ± 8.58%, 75.38% ± 8.32% and 78.36% ± 9.83% of Tregs after co‐culture with cofilin‐1 siRNA‐transfected, scrambled siRNA‐transfected and control mDCs, respectively. There was no significant difference in Treg FOXP3 expression after co‐culture with the different mDCs.

**FIGURE 5:**
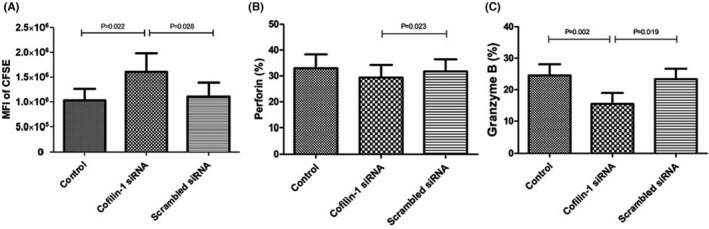
Proliferation of and perforin and granzyme B expression in CD8^+^ T cells co‐cultured with mDCs from patients with SAA after cofilin‐1 siRNA (*n* = 10). (A) The mean fluorescence intensity (MFI) of CSFE in the cofilin‐1 siRNA group was the highest among the three groups, which indicated the lowest proliferation of CD8^+^ T lymphocytes after stimulation with mDCs lacking cofilin‐1. The secretion of perforin (B) and granzyme B (C) by CD8^+^ T lymphocytes decreased significantly in the cofilin‐1 siRNA group

The CSFE MFI of CD8^+^ T lymphocytes was 1,610,313.97 ± 1,182,187.85 when co‐cultured with cofilin‐1 siRNA‐transfected mDCs, and it was higher than that observed after co‐culture with control mDCs (1,030,343.52 ± 742,628.36) and scrambled siRNA‐transfected mDCs (1,107,368.41 ± 90,1731.27). This indicated a decreased proliferative capacity of CD8^+^ T lymphocytes following stimulation by cofilin‐1‐deficient mDCs (Figure 5A).

The perforin expression level was 32.98% ± 17.00%, 29.39% ± 15.51% and 31.71% ± 14.99% in CD8^+^ T lymphocytes incubated with control mDCs, cofilin‐1 siRNA‐transfected mDCs and scrambled siRNA‐transfected mDCs, respectively. The granzyme B expression level in CD8^+^ T lymphocytes was 24.50% ± 11.32%, 15.49% ± 10.89% and 23.35% ± 10.56%, respectively. The perforin and granzyme B expression levels in CD8^+^ T lymphocytes co‐incubated with cofilin‐1 siRNA‐transfected mDCs were significantly lower than those in the other two conditions (*p* = 0.023 and 0.002, respectively; Figure 5B, C).

## 4 DISCUSSION

In patients with SAA, undetermined antigens stimulate an increase in the number and function of mDCs, and this contributes to a Th1/Th2 imbalance and CTL overactivation, leading to pancytopenia.^16^ The dysregulated immune response observed in patients with SAA involves a rapid and potent response by numerous activated and mature immune cells, subsequently carrying out extensive phagocytosis and migration. Moreover, when cells participate in directional migration, the dissociation and aggregation of actin occur simultaneously, causing the cell morphology to change toward the stimulated side. Thus, the cytoskeleton undergoes intense remodelling during this process. The role of the cytoskeletal system, including cofilin, fascin, profilin 1 and other cytoskeletal binding proteins, in autoimmune diseases has been reported.^9^, ^10^ In our previous study, we found that the expression of profilin 1 increases on the mDCs of patients with SAA, and this may affect mDC function.^17^ In the present study, we observed that the expression of cofilin‐1 in mDCs from patients with SAA increased at both mRNA and protein levels. Moreover, cofilin‐1 expression in mDCs from patients with SAA was correlated with their immune indexes and disease severity. After remission, the level of cofilin‐1 decreased to a level similar to that in HCs. Through correlation analysis between the level of cofilin‐1 in mDCs and clinical data of patients with SAA, we found that the level of cofilin‐1 in mDCs of patients with SAA was closely related to the immune status and disease severity in patients with SAA. Therefore, it is plausible that cofilin‐1 in mDCs participates in the immune pathogenesis of SAA. The higher the level of cofilin‐1, the greater the Th1‐CTL immune activation, leading to worsened immunopathology and more severe disease.

Dendritic cells (DCs) are major antigen‐presenting cells in the human body and upstream regulators of SAA immune pathogenesis. Their biological function is to take up, process and present antigens. DC function can be determined by phagocytosis, migration and co‐stimulatory molecule expression. Various studies have focused on the role of cofilin‐1 in DC function. For example, activated cofilin‐1 upregulates phagocytosis in DCs.^11^, ^12^ Additionally, cofilin‐1 promotes the remodelling of the actin cytoskeleton in the formation of a phagocytic cup by accelerating actin turnover, in turn facilitating the formation of phagocytic corpuscles.^18^ Interference with cofilin downregulates the phagocytic function of cells.^11^ New free terminal ends could be generated from F‐actin after cleavage by cofilin‐1, forming DC lamellipodia, which mediate the cell's directional migration to lymphoid tissues.^19^ CD80 and CD86 are markers of mature mDCs and critical surface proteins that provide co‐stimulatory signals to lymphocytes, reflecting the ability of mDCs to stimulate T cells. Dynamic cytoskeleton remodelling has been shown to regulate the expression of antigen‐presenting molecules on the cell surface.^20^ Furthermore, the inactivation of cofilin‐1 decreases the ability of DCs to migrate and co‐stimulate T cells.^13^ To further confirm the influence of increased cofilin‐1 on mDC function in SAA, RNA interference was employed, which successfully silenced cofilin‐1 expression. Thereafter, the functional changes of mDCs were compared between cofilin‐1‐competent and cofilin‐1‐silenced cells. A reduction in the cofilin‐1 level did not affect the proliferation capacity and apoptosis of mDCs. However, the knockdown of cofilin‐1 significantly reduced the phagocytosis and migration of and CD86 expression in mDCs from patient with SAA. A possible underlying mechanism is that the increased cofilin‐1 level in the mDCs of patients with SAA maintain their phagocytic function, allowing greater antigen uptake, processing and presentation, in turn stimulating the immune response. Moreover, this upregulated migratory ability allows the cells to remain in a higher state of activation. Enhanced expression of the co‐stimulatory molecule CD86 promotes immune synapse formation and co‐stimulatory signal transmission, driving the proliferation and differentiation of effector T cells. Cofilin‐1 upregulation may be one of the reasons for enhanced mDC activation in patients with SAA.

Cofilin‐1 functions in the cytoskeletal system through F‐actin remodelling. The inactivation of cofilin‐1 may lead to the abnormal accumulation of F‐actin at the leading edge of mobile cells, and thus, inhibit cell movement.^21^ To verify the mechanism by which cofilin‐1 affects mDCs in patients with SAA, F‐actin in mDCs was labelled with an immunofluorescent stain. Compared with that in the control and scrambled siRNA groups, F‐actin in the cofilin‐1 knockdown group was significantly remodelled, as indicated by an increase in the level of F‐actin and the density of cell processes, presenting a ‘rigid’ state. As the function of cofilin‐1 is to facilitate the dynamic turnover of F‐actin, the knockdown of cofilin‐1 could cause an accumulation of F‐actin and the subsequent dysfunction of mDCs.

Dendritic cells (DCs) play an important role in the proliferation, differentiation and development of CD4^+^ T lymphocytes. The interaction between T cells and DCs relies on immune synapses, and the actin microfilament system ensures interaction between cells. Based on the nature of the activation signals received, DCs control the initiation and differentiation of CD4^+^ T cells into several different phenotypes, including Th1, Th2, Th17 and Tregs.^22^ High‐dose antigenic stimulation could lead to an increased expression of the IL‐12 receptor β chain and induce Th1 differentiation, a process that is affected by the expression of CD80 on DCs.^23^ LKB1 in DCs can modulate the expression of CD86 through phospholipase Cβ1, regulating them to promote Treg differentiation.^24^ Th1 cells mainly produce proinflammatory cytokines,^1^ such as IL‐2, TNF‐α and IFN‐γ, which play major roles in the cellular immune response. Th2 cells secrete IL‐6 and other cytokines, which lead to B lymphocyte activation and antibody production, driving humoral immune responses. Previous studies have shown that although both Th1 and Th2 cells proliferate in patients with SAA, Th1 proliferation is more significant, leading to a shift in the Th1/Th2 balance toward Th1.^25^, ^26^ The levels of the Th1‐related cytokines IL‐2, TNF‐α and IFN‐γ were significantly different among untreated patients with SAA, SAA patients with CR and HCs. Based on the results of the present study, the knockdown of cofilin‐1 inhibits the mDC‐stimulated proliferation of both Th1 and Th2 cells, particularly Th1 cells. This suggests that cofilin‐1 is involved in the stimulation of CD4^+^ T lymphocytes by mDCs, especially Th1 cells. The inhibition of cofilin‐1 in mDCs may reverse the Th1/Th2 imbalance in patients with SAA and achieve the same effect as IST, providing a new target for SAA therapy. FOXP3 is a key molecule in the development, differentiation and maturation of Tregs, as well as for the maintenance of their immunosuppressive function. The expression of FOXP3 was decreased in the Tregs of patients with SAA, leading to a compromised inhibitory function. DCs regulate the homeostasis and immune equilibrium of Tregs.^27^ However, in the current study, there was no evidence of cofilin‐1 affecting mDC‐regulated Treg development.

The expansion and activation of CD8^+^ T lymphocytes are the core of SAA immune pathogenesis. The cofilin‐1/F‐actin system of DCs ensures the controlled activation of T cells, preventing aberrant T‐cell activation‐associated disorders.^28^ Activated CTLs act on the cell membrane to kill target cells in a Ca^2+^‐dependent manner via perforin and granzyme B secretion. The current results revealed that the knockdown of cofilin‐1 in mDCs from patients with SAA reduced the ability of mDCs to stimulate CD8^+^ T lymphocyte proliferation as well as perforin and granzyme B secretion. Therefore, cofilin‐1 may participate in the stimulatory effects of mDCs on CD8^+^ T lymphocytes in patients with SAA.

In summary, elevated cofilin‐1 level may contribute to mDC activation in patients with SAA. The knockdown of cofilin‐1 suppressed phagocytosis, migration and co‐stimulatory molecule expression through remodelling of the actin skeleton, in turn downregulating the activation of effector T cells. Based on the current findings, cofilin‐1 may be a potential biomarker for SAA progression, a promising target for controlling the activation of CTLs in patients with SAA, potentially contributing to the improvement of SAA treatment options.

## AUTHOR CONTRIBUTION

**Yingying Sun:** Data curation (equal); Formal analysis (lead); Resources (lead). **Yu Zhang:** Methodology (equal); Software (lead); Writing – original draft (equal). **Hong Yu:** Data curation (equal); Investigation (equal); Writing – original draft (equal). **Huaquan Wang:** Conceptualization (equal); Supervision (equal); Writing – review & editing (lead). **Zonghong Shao:** Conceptualization (supporting); Funding acquisition (equal); Project administration (supporting). **Chunyan Liu:** Funding acquisition (equal); Project administration (supporting); Writing – review & editing (supporting).

## CONFLICT OF INTEREST

The authors declare that the research was conducted in the absence of any commercial or financial relationships that could be construed as a potential conflict of interest.

## Supporting information

Supplementary Material

## Data Availability

The data that support the findings of this study are available from the corresponding author upon reasonable request.
