# Supplementary material for: Cofilin‐1 participates in the hyperfunction of myeloid dendritic cells in patients with severe aplastic anaemia
Source: J Cell Mol Med. 2022 May 17;26(12):3460–70. doi: 10.1111/jcmm.17359 (PMC9189344; doi:10.1111/jcmm.17359)
Supplement: Supplementary file 1 — Supplementary Material [file JCMM-26-3460-s001.docx]

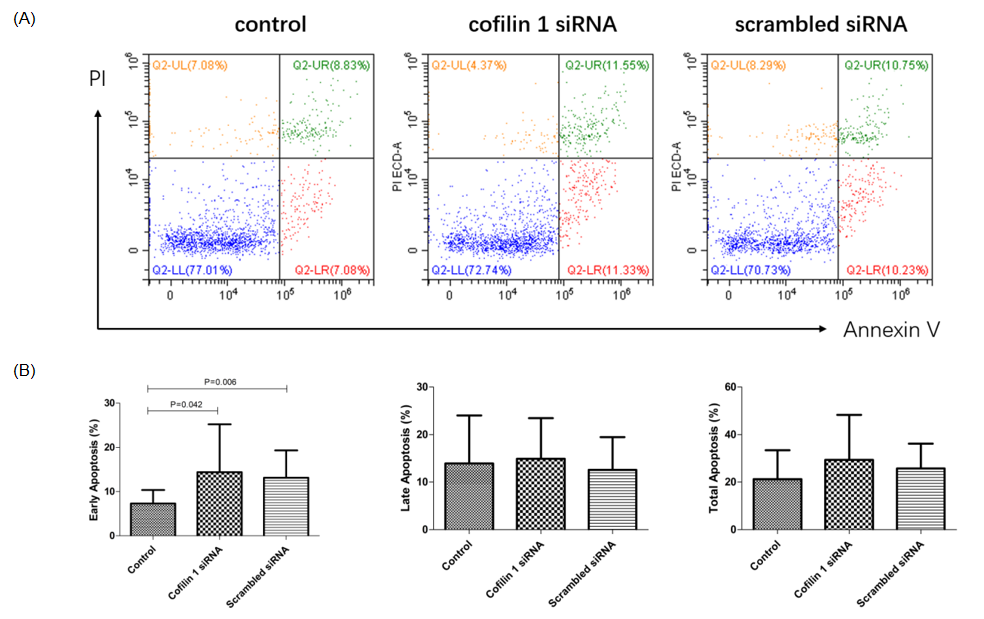


Fig.S1 (A) Apoptosis of mDCs in the control group, cofilin-1 siRNA group and scrambled siRNA group were detected by flow cytometry. (B) Statistical diagram of proportion of early apoptosis, late apoptosis and total apoptosis in control group, cofilin-1 siRNA group and scrambled siRNA group.

Table S1 The proportion of early apoptosis, late apoptosis and total apoptosis in control group, cofilin-1 siRNA group and scrambled siRNA group.

|  | Control | Cofilin 1 siRNA | Scrambled siRNA |
| --- | --- | --- | --- |
| Early Apoptosis(%) | 7.32±3.05 | 14.40±10.82^*^ | 13.15±6.19^*^ |
| Late Apoptosis(%) | 13.92±10.10 | 14.91±8.52 | 12.60±6.86 |
| Total Apoptosis(%) | 21.24±12.20 | 29.31±18.98 | 25.75±10.43 |

^*^Compared with control, P<0.05.
